# Translation, cross-cultural adaption and measurement properties of the evidence-based practice profile

**DOI:** 10.1186/s13104-017-2373-7

**Published:** 2017-01-13

**Authors:** Kristine Berg Titlestad, Anne Kristin Snibsoer, Hilde Stromme, Monica Wammen Nortvedt, Birgitte Graverholt, Birgitte Espehaug

**Affiliations:** 1 Institute of Social Work and Social Education, Faculty of Health and Social Sciences, Western Norway University of Applied Sciences, Bergen, Norway; 2 Centre for Evidence-Based Practice, Faculty of Health and Social Sciences, Western Norway University of Applied Sciences, Bergen, Norway; 3 The Norwegian Institute of Public Health, Oslo, Norway

**Keywords:** Evidence-based practice, Students, Questionnaires, Reliability, Validity, Responsiveness, Psychometrics

## Abstract

**Background:**

The evidence-based practice profile (EBP^2^) questionnaire assesses students’ self-reported knowledge, behaviour and attitudes related to evidence-based practice. The aim of this study was to translate and cross-culturally adapt EBP^2^ into Norwegian and to evaluate the reliability, validity and responsiveness of the Norwegian version.

**Methods:**

EBP^2^ was translated and cross-culturally adapted using recommended methodology. Face validity and feasibility were evaluated in a pilot on bachelor students and health and social workers (*n* = 18). Content validity was evaluated by an expert panel. Nursing students (*n* = 96), social educator students (*n* = 27), and health and social workers (*n* = 26) evaluated the instrument’s measurement properties. Cronbach’s alpha was calculated to determine internal consistency. Test–retest reliability was evaluated using the intra-class correlation coefficient (ICC) and standard error of measurement (SEM). Discriminative validity was assessed by independent sample *t* test. A confirmatory factor analysis (CFA) was performed to assess the structural validity of a five-factor model (*Relevance*, *Sympathy*, *Terminology*, *Practice* and *Confidence*) using the comparative fit index (CFI) and the root mean square error of approximation (RMSEA). A priori hypotheses on effect sizes and *P* values were formulated to evaluate the instrument’s responsiveness.

**Results:**

The forward–backward translation was repeated three times before arriving at an acceptable version. Eleven of 58 items were re-worded. Face validity and content validity were confirmed. Cronbach’s alpha was 0.90 or higher for all domains except *Sympathy* (0.66). ICC ranged from 0.45 (*Practice*) to 0.79 (*Terminology*) and SEM from 0.29 (*Relevance*) to 0.44 (*Practice*). There was a significant mean difference between exposure and no exposure to EBP for the domains *Relevance*, *Terminolog*y and *Confidence.* The CFA did not indicate an acceptable five-factor model fit (CFI = 0.69, RMSEA = 0.09). Responsiveness was as expected or better for all domains except *Sympathy*.

**Conclusions:**

The cross-culturally adapted EBP^2^-Norwegian version was valid and reliable for the domains *Relevance*, *Terminology* and *Confidence*, and responsive to change for all domains, except *Sympathy*. Further development of the instrument’s items are needed to enhance the instruments reliability for the domains *Practice* and *Sympathy*.

## Background

Evidence-based practice (EBP) is embedded in health policy and healthcare professionals are increasingly expected to inform their practice by evidence [1]. EBP is a systematic approach for making clinical decisions where current best available research evidence is integrated with clinical experience and patient preferences, within a context of available resources [2]. This involves the five steps model of EBP: asking clinical questions, searching for and appraising research evidence, integrating the evidence into clinical practice and evaluating performance [3]. However, the implementation of EBP is deficient and there is a gap between best practice and delivered health care [4]. Lack of training is one barrier for implementing EBP [4–6].

EBP training was initially focused on upskilling healthcare professionals within the health workplace [7, 8]. Increasingly, the awareness of EBP teaching among undergraduate students has grown [9, 10]. An international curriculum framework for EBP and recommendations for EBP teaching and education have been described in the Sicily consensus statement on EBP [2]. This consensus statement recommends that teaching in EBP should be grounded in the five step model of EBP. Another recommendation is that EBP should be a basic and essential component of healthcare curricula [2, 11].

The integration of EBP in undergraduate healthcare education requires instruments to assess EBP competence and performance [12]. However, systematic reviews over such tools have mostly identified instruments developed for healthcare professionals and medical students [9, 12–14]. In addition, a limited number of instruments have established measurement properties [12, 13, 15] and few measure all five steps of EBP [12, 14, 15].

The evidence-based practice profile (EBP^2^) questionnaire, is a tool that assesses EBP knowledge, attitudes and behaviour among healthcare students [7]. It was developed in Australia by McEvoy et al. [7] and validated for students and healthcare professionals in different healthcare disciplines. The EBP^2^ is a self-reported instrument with acceptable measurement properties. It was the only identified tool that examined the principles of EBP and the five steps of EBP, and suitable for measuring EBP across health professions. The aim of this study was to translate and cross-culturally adapt EBP^2^ into Norwegian and to evaluate the reliability, validity and responsiveness of the Norwegian version.

## Methods

We translated and cross-culturally adapted the EBP^2^ questionnaire into Norwegian following recommended methodology [16–18]. The consensus-based standards for the selection of health measurement instruments (COSMIN) checklist was used as a framework to guide our choices of measurement properties and parameters [19, 20].

### The original instrument

EBP^2^ was originally composed by collating characteristics of EBP from previous existing self-report questionnaires, identified by a systematic review of the literature [7]. The measurement properties were tested across a range of health professionals, academics, and students within health or non-health background. The questionnaire consists of 74 items, 58 domain items and 16 non-domain items. In addition, 13 items address the respondents’ demographic characteristics. The respondents indicate their scores on a 5-point Likert scale, and the questionnaire takes 10–12 min to complete.

An exploratory factor analysis (EFA) revealed the presence of the five domains *Relevance, Sympathy, Terminology, Practice* and *Confidence* [7]. *Relevance* (14 items) refers to the value, emphasis and importance placed on EBP, *Sympathy* (7 items) refers to the individual’s perception of the compatibility of EBP with professional work, *Terminology* (17 items) refers to the understanding of common research terms, *Practice* (9 items) refers to the use of EBP in clinical situations and *Confidence* (11 items) refers to the perception of an individual’s ability with EBP skills [7, 21]. The instrument is multidimensional with each domain score calculated as the sum of all items in each domain, and each item weighted equally. The domain of Sympathy is negatively keyed [7].

The EBP^2^ measurement properties confirmed good internal consistency and test–retest reliability [7]. Convergent validity was tested by comparing EBP^2^ to the 24-item Upton & Upton questionnaire [22]. The Upton & Upton questionnaire covered three of the five factors in EBP^2^ (*Practice*, *Confidence* and *Sympathy*) and the EBP^2^ questionnaire demonstrated good convergent validity for the three comparable factors [7]. The EBP^2^ distinguished between groups exposed to EBP and unexposed groups for three of the domains (*Relevance, Terminology* and *Confidence*) [7].

### Translation and cross-cultural adaption process

Permission to translate the EBP^2^ into Norwegian was granted from the copyright holder. Following recommended methodology [16–18], two bilingual translators (KBT, HS), with expertise in the construct measured and whose native language was Norwegian, translated the questionnaire independently of each other. The translators aimed at a conceptual and cultural equivalence, rather than a word-for-word translation. The forward translations were reviewed and discussed by an expert panel that consisted of a professor in EBP (MWN), an assistant professor (AKS) and a master student (KBT). Translators and members of the expert panel were fluent in both Norwegian and English.

The expert panel agreed on a version for back-translation. A professional translator (SG), whose native language was English, performed the back-translation. SG had no knowledge about the original instrument. Discrepancies between the back-translation and the original version were discussed with the copyright holder. The forward–backward translation process was repeated three times until an acceptable version was agreed upon by the expert panel and the copyright holder.

We pilot tested the comprehension of the translated version of EBP^2^ on 18 participants from five different health and social professions (Table 1). Nine of these participants were considered experts in EBP. All participants completed the questionnaire while they read aloud the item response options and their own choice of answer. After completion, the participants were interviewed by KBT to elaborate on items or response options that were unclear. The data from the interviews were organised and summarised using “The Problem Respond Matrix” [23]. The Problem Respond Matrix was developed to standardise the analysis of cognitive data and can be used to identify items that are unclear to respondents.

**Table 1:** Characteristics of participants in the pilot test (*n* = 18)

	n	%
Gender		
Male	1	6
Female	17	94
EBP training		
None	2	11
3–10 h	2	11
10–20 h	5	28
More than 20 h	9	50

Profession	Students		Professional	
	n	%	n	%
Nurse	3	17	4	22
Social educator	2	11	4	22
Physiotherapist	0	0	3	17
Occupational therapist	0	0	1	5.6
Social worker	0	0	1	5.6

*n* number of cases

### Evaluation of measurement properties

#### Participants and data collection

The total number of eligible participants was 247, representing bachelor students in nursing (*n* = 152) and social education (*n* = 63) from a large University College in Norway, and health and social workers from a local hospital (*n* = 32). Second year nursing students attending an EBP course, were recruited to evaluate the questionnaire’s responsiveness. The EBP course was equivalent to 5 ECTS credits (The European Credit Transfer and Accumulation System) [24] and emphasised the acquisition of knowledge and skills in the principles of EBP and the five-step EBP model. The 3-week course was clinically integrated and students were formally assessed at the end of the course. Second year social educator students attending a course without EBP exposure and clinical health and social workers from a dayshift were enrolled to evaluate test–retest reliability.

The bachelor students were recruited at the start of a classroom session and the health and social workers at a shift handover. Data were collected from January to April 2014. The questionnaire was answered twice by all participants with a time interval of 3 weeks for the test–retest evaluation among social educator students and health and social workers, and with a time interval of 4 weeks for the responsiveness evaluation among nursing students. The test conditions were similar at both measurement times. The questionnaires were administered independently of each other. Participants who answered the questionnaire twice and had less than 25% missing items were included.

#### Statistical analysis

Statistical analyses were performed using *IBM SPSS Statistics* version 22 [25] and *R* [26]. As in the evaluation of the original EBP^2^ only domain items were included in the analyses [7]. The level of significance was set at 0.05. Respondents with more than 25% missing values were excluded from all analyses, following the procedure reported by McEvoy et al. [7]. Respondents with more than 20% missing values in one domain were excluded from analysis of that specific domain.

Reliability was assessed by internal consistency, test–retest reliability and measurement error. For internal consistency, Cronbach’s alpha was applied for every domain and was considered good between 0.70 and 0.90 [17]. Intraclass correlation coefficient (ICC) determined the test–retest reliability (intra-rater reliability), using a two-way random model, absolute agreement. ICC was calculated for each item and each domain, and ICC > 0.70 was deemed acceptable [27]. Cohen’s linear-weighted kappa was calculated for each item. Minimum acceptable kappa value was 0.60, while values of 0.75 or higher were considered good [24, 28]. Measurement error was expressed as standard error of measurement (SEM) using the formula SEM = SD/√2. The larger the SEM, the lower the test reliability and the less precision in the measures taken and scores obtained [17].

Discriminative validity for levels of EBP exposure was assessed by independent sample *t* test. Measurements obtained from the nursing students after participation in a course in EBP (5 ECTS) were compared to re-test measurements among social educator students and health and social workers without this course. Structural validity was assessed by factor analysis. Confirmatory factor analysis (CFA) was performed to test whether the data fit the original five-factor structure. To evaluate model fit we used the comparative fit index (CFI), the root mean square error of approximation (RMSEA) and the standardized root mean square residual (SRMR). Guidelines suggest that models with CFI close to 0.95 or higher, RMSEA close to 0.06 or lower and SRMR close to 0.08 or lower represent a good-fitting model [29].

We formulated a priori hypotheses on Effect Size (ES) and Paired *t* test results (*P* value) to measure the questionnaire’s responsiveness. Based on the cohort of Long et al. [30], we hypothesized a smaller ES in our study due to our 3-week course as opposed to 13-weeks in Long et al. Thus, we hypothesized ES to be larger than moderate at *Relevance*, larger than small at *Sympathy*, larger than moderate at *Terminology*, less than small at *Practice* and larger than small at *Confidence*. ES was considered large if 0.8, moderate if 0.5 or small if 0.2 [31]. We expected no change in the ES for the domain *Practice,* as participants were asked about EBP activities in the past year.

## Results

### Translation and cross-cultural adaption

The forward–backward translation was repeated three times before arriving at an acceptable version. “The Problem Respond Matrix” showed that eleven items were unclear or challenging to understand (the matrix is available on request). These items were re-worded after consulting the copyright holder.

The pilot participants with expertise in EBP (*n* = 9) confirmed face validity. The expert panel assessed content validity and found the questionnaire, questions and rating scale clinically reasonable and relevant to the area of applicability. The layout of the EBP^2^-Norwegian version is similar to the original with the same number of items and demographic questions.

### Evaluation of measurement properties

**Fig. 1:**
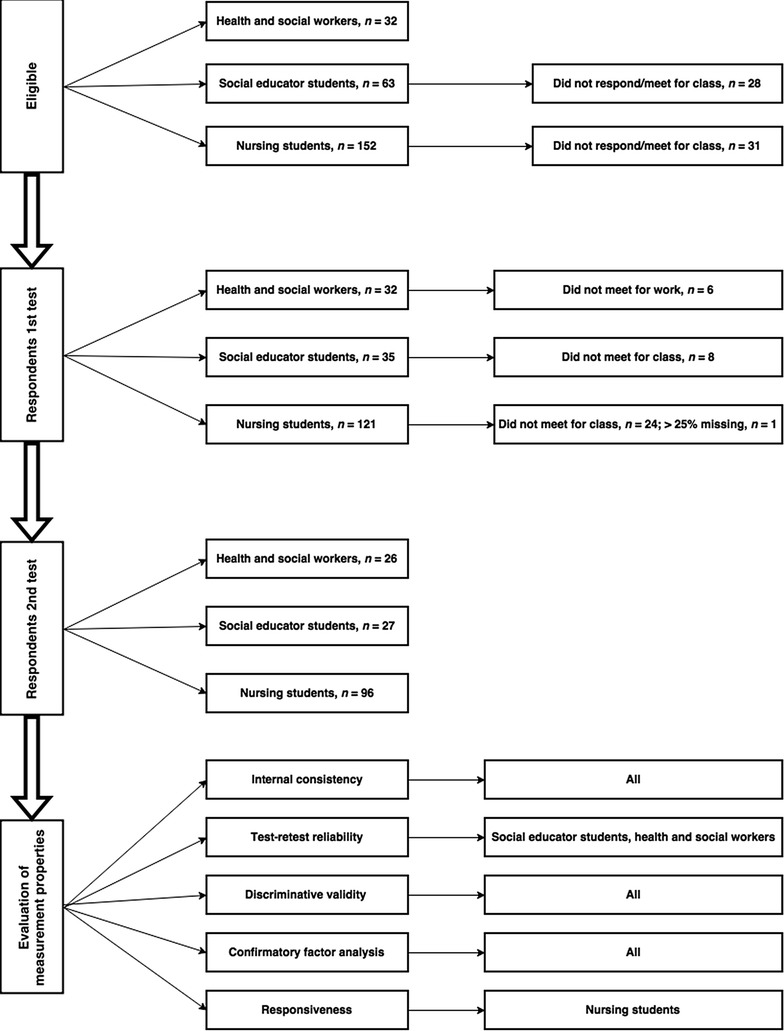
Flowchart describing the process of the assessment of measurement properties

A total of 247 individuals were eligible for participation. Among the eligible students (*n* = 215), 188 (87%) met for the first teaching session and answered the questionnaire. The study included 149 participants responding at both measurements: 96 nursing students testing the questionnaire’s responsiveness, and 27 social educator students and 26 health and social workers testing test–retest reliability (Fig. 1). We excluded participants who did not meet for the retest (*n* = 38) and respondents with more than 25% missing items (*n* = 1).

Most of the participants were females (87%). The mean age was 28.2 years (range 20–61) (Table 2). The average number of items with missing values was 0.7 (SD = 0.9) per participant. No items had more than 2.7% missing values.

**Table 2:** Characteristics of participants

Characteristics	All (n = 149)		Test–retest reliability (n = 53)		Responsiveness (n = 96)	
	n (%)	Mean (SD)	n (%)	Mean (SD)	n (%)	Mean (SD)
Age	148 (99)	28.2 (10.5)	53 (100)	35 (12.7)	95 (99)	24.4 (6.4)
Gender						
Male	19 (13)		6 (11)		13 (14)	
Female	130 (87)		47 (89)		83 (87)	
English						
Easy	71 (48)		23 (43)		48 (50)	
Neither hard nor easy	62 (42)		22 (42)		40 (42)	
Hard	11 (7)		5 (9.4)		6 (6)	
Very hard	1 (0.7)		1 (1.9)		0 (0)	
Missing	4 (2.7)		2 (3.8)		2 (2)	
Profession^a^						
Nurse			23 (88)			
Occupational therapist			1 (4)			
Social educator			2 (7.7)			
EBP training^a^						
None			17 (65)			
3–10 h			3 (12)			
10–20 h			2 (7.7)			
More than 20 h			2 (7.7)			
Missing			2 (7.7)			

*n* number of cases, *SD* standard deviation

^a^Among the included health and social workers (*n* = 26)

Cronbach’s alpha for the five domains ranged from 0.49 (*Sympathy*) to 0.92 (*Terminology*) on the first test. On the second test, Cronbach’s alpha ranged from 0.66 (*Sympathy*) to 0.94 (*Terminology* and *Confidence*) (Table 3).

**Table 3:** Internal consistency (*n* = 149)

Domain	*N* of items	1st test		2nd test	
		n	Cronbach’s alpha	n	Cronbach’s alpha
Relevance	14	145	0.88	144	0.91
Sympathy	7	144	0.49	145	0.66
Terminology	17	139	0.92	135	0.94
Practice	9	144	0.82	142	0.90
Confidence	11	147	0.91	143	0.94

*n* number of cases

Table 4 shows the results from the analyses of test–retest reliability. ICC ranged from 0.45 (*Practice*) to 0.79 (*Terminology*). Linear-weighted kappa for single items ranged from −0.02 (*Sympathy*) to 0.68 (*Terminology*) and SEM values varied from 0.29 (*Relevance*) to 0.44 (*Practice*).

**Table 4:** Test–retest reliability of the questionnaire (*n* = 53)

Domain	Range items weighted kappa’s	Range items ICC	n	Domain ICC (95% CI)	Mean difference	SEM
Relevance	0.25 to 0.54	0.32–0.70	53	0.69 (0.47–0.82)	0.19	0.29
Sympathy	−0.02 to 0.40	0.01–0.50	52	0.47 (0.19–0.63)	0.13	0.32
Terminology	0.28 to 0.68	0.27–0.84	52	0.79 (0.66–0.87)	−0.04	0.36
Practice	0.09 to 0.47	0.02–0.54	53	0.45 (0.21–0.64)	−0.15	0.44
Confidence	0.31 to 0.57	0.41–0.74	53	0.76 (0.62–0.85)	0.00	0.38

*n* number of cases, *CI* confidence interval

There was a significant mean difference between exposure and no exposure to EBP for the domains *Relevance*, *Terminolog*y and *Confidence* (Table 5). The CFA showed that the CFI of the entire model was 0.59 on the first test and 0.69 on the second test. Its RMSEA was 0.090 (95% CI 0.085–0.094) and 0.089 (95% CI 0.084–0.094) while the SRMR was 0.098 and 0.095.

**Table 5:** Discriminative validity for participants with (*n* = 96) and without (*n* = 53) EBP course (5 ECTS points)

Domain	EBP course		No EBP course					
	n	Mean (SD)	n	Mean (SD)	Mean difference	95% CI	*P* value	Effect size (Cohen’s d)
Relevance	96	58.7 (6.5)	53	53.3 (7.5)	5.39	3.05 to 7.73	<0.001	0.76
Sympathy	96	20.3 (3.0)	53	20.3 (3.1)	0.02	−1.06 to 1.07	1.0	0.01
Terminology	96	51.6 (11.9)	53	39.1 (13.3)	12.51	8.32 to 16.71	<0.001	0.99
Practice	96	21.2 (5.8)	53	21.4 (5.3)	−0.19	−2.10 to 1.70	0.84	0.04
Confidence	96	33.8 (8.3)	53	28.8 (8.4)	4.97	2.14 to 7.80	0.001	0.59

*n* number of cases, *SD* standard deviation, *CI* confidence interval

Statistically significant mean differences comparing pre- and post-EBP course measurements were observed for all domains except *Sympathy*. ES values were as expected or better for the domains *Relevance*, *Terminology*, *Practice* and *Confidence*, but lower for *Sympathy* (Table 6).

**Table 6:** Responsiveness of the domain scores of EBP^2^-Norwegian version (*n* = 96)

Domain	*n*	Pre	Post	Pre-post			
		mean (SD)	mean (SD)	mean difference	95% CI	*P* value	Effect size (Cohen’s d)
Relevance	95	54.1 (7.0)	58.6 (6.5)	4.53	3.15 to 5.92	<0.001	0.67
Sympathy	93	20.2 (2.1)	20.2 (3.0)	0.09	−0.73 to 0.56	0.79	0.03
Terminology	96	41.8 (11.9)	51.6 (11.9)	9.81	7.87 to 11.76	<0.001	0.82
Practice	96	19.7 (5.7)	21.2 (5.8)	1.53	0.43 to 2.64	0.007	0.27
Confidence	96	27.0 (8.1)	33.8 (8.3)	6.77	5.36 to 8.19	<0.001	0.83

*n* number of cases, *SD* standard deviation

## Discussion

The EBP^2^ was translated and cross-culturally validated into Norwegian, using acknowledged standards. The EBP^2^-Norwegian version was found to be a reliable tool for measuring three of the five domains, namely *Relevance*, *Terminology* and *Confidence.* Further, the EBP^2^-Norwegian version was able to detect a change after EBP exposure in all domains, except for *Sympathy*. Content validity was established. Discriminative validity was verified for *Relevance*, *Terminology* and *Confidence*, but structural validity did not confirm the original five-factor model.

In our study, the domain *Sympathy* revealed low reliability and poor responsiveness. In the evaluation of the original EBP^2^ the measurement properties were also poorest for *Sympathy*, although with better results [7]. While this domain consists of the smallest number of items, a likely explanation for inadequate internal consistency may be poor interrelatedness among the items for this domain. Furthermore, *Sympath*y consists of negatively worded items with reversed scores. Although reversed score items serve the useful function to disrupt undesirable response sets, they may confuse respondents if the altered direction of the wording goes unnoticed [32]. In addition, the negatively worded items were more challenging to translate than the others, and it might be that the Norwegian translation did not fully capture the English phrasing.

Test–retest reliability was low for the domains *Sympathy* and *Practice*. It is possible that the inconsistency we observed relates to raised EBP consciousness between the measurement periods, through exposure to questions, reflection and better understanding [33]. However, both domains refer to the use of EBP in clinical situations and the compatibility of EBP with professional work [7]. They rely on an understanding of EBP concepts and day-to-day practical incorporation of EBP, and it may be that the inconsistency we found reflects the homogeneity in our sample and its diverse familiarity with EBP concepts. A further exploration with a larger and more heterogeneous sample could determine if prerequisite EBP acquaintance is essential to fully understand the questions. Nonetheless, the results from the test–retest reliability analyses may be used to shape item-retention decisions, by performing analyses of the items’ ICC values, refining item wording with the target population through cognitive interviews and asking an expert panel to consider content validity [33]. A review for potential cultural, contextual, translational and interpretational limitations of the items on the EBP^2^-Norwegian version, with emphasis on the domains *Sympathy* and *Practice* is essential.

Norwegian health and social workers with experience in EBP confirmed face and content validity on the EBP^2^-Norwegian version. As the original scale, the EBP^2^-Norwegian version discriminated between low and high exposure of EBP for *Relevance*, *Terminology* and *Confidence*. Moreover, the number of missing items was low and did not indicate problems with the instrument, like incomprehension or a poor fit between answers and response options [17]. This suggests that the participants found the EBP^2^-Norwegian version feasible. Still, the CFA did not confirm the original five-factor model.

As hypothesized, the domains most likely to be affected by the 3 week EBP course were *Relevance* and *Terminology*. For these domains, ES was larger than expected. In addition, we observed a larger change in ES for *Confidence* than predicted. We hypothesized a smaller ES than observed by Long et al. [30], since our students participated in a 3-week EBP course and the students in the previous study received a 13-week EBP course. Interestingly, the EBP course in our study fulfills the recommendations for EBP teaching, like clinical integration, multifacteted teaching strategies and formal assessment [34]. It is possible that we underestimated the value of these important aspects when we formulated the a priori hypotheses on ES.

One strength of this study is the application of recommended frameworks [16, 35] to guide a transparent translation, cross-cultural adaption, evaluation and reporting of measurement properties. Our sample size was adequate for evaluation of internal consistency, test–retest reliability, discriminative validity and responsiveness. Still, according to de Vet [17] there should be a minimum of 100 participants, but preferably four to ten participants per item to perform a satisfactory CFA [36]. Our sample size of 149 participants may therefore be too small for valid fit measures in the CFA analysis. Furthermore, bachelor students from two different health and social studies programmes were included in the Norwegian study, compared to students from five different health programs in the Australian study [7]. A larger, more heterogeneous sample could have improved the methodological information of the five-factor model.

To assess EBP competence in all five EBP steps with one instrument is a challenge [15, 37]. Self-reported competence in EBP may cause respondents to over-estimate their actual competence [38], and the most common way to measure EBP learning has been to evaluate attitudes and self-efficacy with self-reported instruments [15]. According to the CREATE framework (classification rubric for EBP assessment tools in education) actual EBP knowledge, skills and behaviour need to be assessed through cognitive testing, performance assessment and activity monitoring [15]. Hence, the limitations of the EBP^2^ tool should ideally be triangulated with additional information gained from instruments assessing actual knowledge and skills.

EBP education is increasingly common across clinical settings and higher educational programmes. Still, the possibility to measure the impact of EBP education has been limited to a few validated tools. With the cross-cultural adaption and measurement evaluation of the EBP^2^-Norwegian version our study adds knowledge to this subject.

## Conclusions

The measurement properties of EBP^2^-Norwegian version was reliable and valid for the domains *Relevance, Terminology* and *Confidence.* Further research is needed to appraise the domains *Sympathy* and *Practice.* We recommend further studies of EBP^2^-Norwegian version with a larger and more heterogeneous sample. We also recommend further linguistic improvement of the questionnaire by using the results from testing test–retest reliability to shape the item-retention decisions.

## Abbreviations

EBP: evidence-based practice
EBP^2^: the evidence-based practice profile
COSMIN: the consensus-based standards for the selection of health measurement instruments
EFA: exploratory factor analysis
KBT: Kristine Berg Titlestad
HS: Hilde Stromme
MWN: Monica Wammen Nortvedt
AKS: Anne Kristin Snibsoer
SG: Simon Goudie
ECTS: the European credit transfer and accumulation system
ICC: intraclass correlation coefficient
SEM: standard error of measurement
CFA: confirmatory factor analysis
CFI: comparative fit index
RMSEA: root mean square error of approximation
SRMR: standardized root mean square residual
ES: effect size
NSD: the Norwegian social science data services
CREATE: the classification rubric for EBP assessment tools in education
